# ‘Vulnerable Monsters’: Constructions of Dementia in the Australian Royal Commission into Aged Care

**DOI:** 10.1007/s11196-023-09979-w

**Published:** 2023-03-14

**Authors:** Kristina Chelberg

**Affiliations:** Australian Centre for Health Law Research, School of Law, Faculty of Law and Business, Queensland University of Technology, GPO Box 2434, Brisbane, QLD 4001 Australia

**Keywords:** Dementia behaviours, Aged care, Vulnerability, Monstrous, Abjection, Royal Commission Aged Care, Restrictive practices, Wandering

## Abstract

This paper argues that while regulatory frameworks in aged care authorise restraints to protect vulnerable persons living with dementia from harm, they also serve as normalising practices to control challenging monstrous Others. This argument emerges out of an observed unease in aged care discourse where older people living with dementia are described as ‘vulnerable’, while dementia behaviours are described as ‘challenging’. Using narrative analysis on a case study from the Final Report of the Australian Royal Commission into Aged Care Quality and Safety (RCAC), this paper investigates how the RCAC (re)produced constructions of persons with dementia as ‘vulnerable monsters’. Drawing upon monstrous theory about ‘unruly and leaky’ bodies, extracts from the case study reveal how the RCAC repeated and reinforced monstrous constructions of dementia. Dementia behaviours, particularly ‘wandering’, were constructed through a dehumanising crisis frame that produced ‘challenging’ bodies and legitimised ‘last resort’ normalising practices, such as physical and chemical restraints. In failing to resist monstrous constructions of dementia behaviours, the RCAC accepted and authorised a regime of scaled responses leading to restrictive practices for control of challenging bodies in aged care. Although dementia care and restrictive practices received substantial attention in the RCAC, this paper reveals a missed opportunity for deeper review of institutionalised use of restraints that has relevance for ongoing reform of Australian aged care following conclusion of the RCAC.

## Introduction

The recent Australian Royal Commission into Aged Care Quality and Safety (RCAC) identified dementia care and restrictive practices as key issues for reform of the Australian aged care system. Debates about restrictive practices in dementia and aged care take place within Australian aged care public and media discourse, where older people living with dementia in institutional aged care are often described as one of the ‘most vulnerable’ groups in society in need of protection.^1^ At the same time, bodies of older persons living with dementia who exhibit dementia behaviours^2^ are described as ‘challenging’ and ‘disruptive’ and the target of authorised ‘management’ strategies, such as chemical and physical restraint. This discursive tension filtered into the Final Report of the RCAC where older people living with dementia were recognised as a ‘vulnerable’ group,^3^ while their bodies were problematised in discussions about their ‘challenging’ behaviours.^4^ However, while excessive use of restrictive practices received substantial attention by the RCAC, its focus was an examination of the medico-legal framework of clinical justification and legal validity of restraints. Taking this as a starting point, this paper queries the RCAC’s emphasis on the *regulation* of authorised use, rather than the *role*, of restrictive practices for dementia behaviours within institutional aged care. Building on the work of Shildrick and Kristeva, this paper explores the RCAC’s negotiation of a troubled hybrid conception of persons living with dementia as both ‘vulnerable’ and ‘challenging’ that produced a monstrous Other at this discursive border and frames ambivalent law and policy about restrictive practices in aged care.

Evidence for this paper emerged from findings of a narrative analysis of the Garden View Case Study (Case Study) documented by the RCAC in its final report. The Case Study records the experience of Mr Terance Reeves, a man living with dementia, who was subjected to physical and chemical restraints at Garden View Nursing Home, which made it an appropriate text to explore constructions of dementia by the RCAC. As it draws together several concepts about vulnerability and monstrousness to build its final argument about restrictive practices in dementia aged care, it is worthwhile signposting this paper is structured into five sections. The first section introduces the RCAC and restrictive practices in dementia aged care, while the second situates it within existing scholarship on monstrous co-constructions of persons as vulnerable and challenging, ‘wandering’ and ‘last resort’ responses. The third section sets out RCAC’s approach to restrictive practices in dementia care and the facts of the Case Study, and the fourth describes three key narratives about ‘challenging’ dementia behaviours, ‘wandering’ and ‘last resort’ forms of medio-legal control. This leads to discussion in the fifth section about the use of restrictive practices as an authorised normalising practice for persons living with dementia within institutional aged care.

Before turning to the first section, this article does not deny the hard realities of dementia care practices for dementia behaviours nor legal tensions relating to decision-making and consent for persons with cognitive impairment. Instead, its aim is to make explicit ‘on what kinds of assumptions, what kinds of familiar, unchallenged, unconsidered modes of thought the practices that we accept rest’ [12: 154]. This approach invites deeper understanding of the problem of vulnerable people with challenging behaviours in institutional care that leads to use of restrictive practices and has wider relevance in cultures of disability and aged care.

## Royal Commission into Aged Care

This section begins by describing the context of the (over)use of restrictive practices for dementia behaviours within institutional aged care as a matter of inquiry referred to the RCAC [13]. This leads to an outline of the formation of the RCAC, its recommendations for ‘transformational’ aged care reforms and government implementation of measures to address substandard dementia care and excessive use of restrictive practices. It concludes by acknowledging a growing field of literature examining Royal Commissions as a subject of critical legal scholarship.

### Dementia and Restrictive Practices

Dementia care and restrictive practices remain unresolved issues in contemporary institutionalised aged care. On the basis a majority of persons in institutional aged care hold a diagnosis of dementia and management of dementia behaviours is a significant issue within institutional care, issues about overuse of physical and chemical restraints often reflect issues in dementia care.^5^ Physical restraints include the use of lap belts, deep chairs, removal of mobility aids or confinement to ‘locked’ or ‘specialised’ dementia units, while chemical restraints include restricted prescription medications such as sedatives, opioids and psychotropics [15: 2–3].^6^ National standards to minimise the use of restrictive practices have been criticised for exempting use of chemical restraints for ‘treatment’ purposes and weak requirements for informed consent [17]. Physical and chemical restraints are associated with physical, emotional and cognitive side effects and have limited effectiveness in managing behaviour, while alternative strategies include environmental, physiological, psychosocial factors, as well as relational care approaches [15: 5–7]. Restrictive practices in institutional aged care operate in Australia under a regulatory system of State and Commonwealth legislation, health practitioners, and medicines, and are complicated by legal tensions in consent and decision-making for persons with cognitive impairment. Additionally, closed aged care facilities and ‘secure’ specialist dementia are argued to operate as places of civil detention that contravene human rights obligations [18, 19].

### Recommendations and Response

The RCAC was established in October 2018 and commenced public hearings in January 2019. The RCAC produced three reports: an interim report ‘Neglect’ in October 2019, a special report on COVID-19 in aged care in October 2020, and a final report ‘Care Dignity and Respect’ in February 2021.^7^ The RCAC was the culminated response to media exposure of institutional aged care failures, successive government enquiries and persistent public calls for reform of the Australian aged care system.^8^ Under its terms of reference, the RCAC was to inquire into the ‘extent of substandard care being provided, including mistreatment and all forms of abuse’ as well as the ‘increasing number of Australians living with dementia, having regard to the importance of dementia care for the future of aged care services’, in addition to ‘positive behaviour supports to reduce or eliminate the use of restrictive practices’ [13].

In its Final Report, the RCAC made 148 recommendations for substantial reform of the Australian aged care system, including dementia-specific proposals for improved support pathways, specialist services, residential design, access to allied and mental health, staff training in dementia, as well as greater representation for people living with dementia in the aged care system. The RCAC concluded provision of dementia care is often substandard and associated with excessive use of restrictive practices by aged care providers [22: 100]. Detailed consideration was given by the RCAC to ‘eliminating and reducing’ restrictive practices on the basis their ‘overuse’ was a ‘major quality and safety issue’ [8: 108]. The RCAC stated restrictive practices ‘impact the liberty and dignity’ of persons in aged care, raise ‘fundamental human rights questions’ and, if applied without consent, breach civil or criminal laws [8: 110]. Whilst a ‘strong and effective regulatory framework’ is needed, the RCAC acknowledged regulation alone would not eliminate restraints and advocated systemic reform across a range of recommendations designed to ‘change the approach to restrictive practices in aged care’ [8: 111–115]. The ‘new’ approach to restrictive practices was to be guided by respect and support for ‘people’s rights, dignity and personal autonomy’ and balanced with ‘clarity about the circumstances in which … restrictive practices, may be authorised’ [22: 93]. Specifically, the RCAC proposed a strict framework to regulate restrictive practices including alternative strategies, independent expert assessment, behaviour support plans, informed consent, monitoring, reporting and penalties for breach, where any exception was to apply only as ‘long as needed to prevent significant harm’ [22: 93].^9^

In response to the RCAC’s recommendations, a number of legislative reforms have been (or will be) introduced, including provision for consumer quality ratings of residential facilities, aged care provider code of conduct, and changes to funding models and serious incident reporting.^10^ In relation to restrictive practices, legislative obligations now require aged care providers to minimise ‘unregulated and unwarranted’ use of restrictive practices and obtain informed consent on behalf of a person without legal capacity prior to the use of restrictive practices.^11^

### RCAC as Discourse

As an apparatus of executive government within the Westminster tradition, Royal Commissions of Inquiry present a research opportunity as a form ‘official discourse’ [25] or ‘schemes of legitimisation’ [26].^12^ There is an emergent body of critical legal literature on the role of Royal Commissions to address legal and cultural change in diverse fields such as financial services [28–30], genetic modification [31], child sexual abuse [32, 33] and colonisation practices [34, 35]. Following delivery of the RCAC’s Final Report, research has studied media analysis of representations of aged care depicting themes of isolation and marginalisation, abuse and neglect [36]; moral disengagement by aged care staff in institutional care failures [37]; and criminal liability for systemic organisational failures to prevent harms [38].^13^ However, what has not been the subject of scrutiny is the *role*, rather than *overuse*, of restrictive practices in institutional aged care that frame law and regulation related to the use of physical and chemical restraints. This paper builds on dementia, feminist and disability scholarship to explore the ways the figure of the monster is constructed by the aged care system — and (re)produced by the RCAC — in dementia and dementia behaviours that legitimises use of restraints and medication.

## Monstrous Dementia Discourses

Drawing on existing literature about co-constructions of ‘vulnerable’ people with ‘challenging’ bodies, this section outlines how this discursive tension reflects and reinforces figuration of persons living with dementia as monsters and legitimises forms of control for dementia behaviours. Disability, feminist and dementia scholarship is brought together to establish the framework for analysis of the Case Study. It begins by reviewing disability scholarship about concepts of vulnerability and challenging (abjection) to show that, despite apparent incongruence, both are recognised as representative of monstrous bodies. This leads to an examination of dementia literature about ‘wandering’ as problematised movement in persons with dementia that itself signifies the monstrous. It concludes by narrowing in on literature on ‘last resort’ emergency restrictive practices where monstrous bodies with disability (and dementia) are constructed as an ‘emergency’ that legitimise ‘normalising practices’ within institutional care.

Monsters are unfathomable transgressive beings that defy categories, signal crisis and are ‘always a linguistic and cultural construction’ [42: 58]. Monster theory, with origins in literature and psychoanalysis, has broad application to disability and feminist research, and in law scholarship to explore legal responses to human difference [43–45]. In a monster reading of the Australian Royal Commission into Institutional Responses to Child Sexual Abuse (RCIRCSA), Crofts described the unsettling hybrid nature of the monstrous Other:

Monsters generate fear and fascination because they not only break rules and cross borders, but also challenge the border itself, by being both and neither one thing and another. Monsters resist and refuse easy categorization. They are disturbing hybrids … they are neither/both dead and alive. …the monstrous is produced at the border [46: 128-129].^14^

Where the ‘excessiveness’ of monstrous bodies place normative categories in crisis and require ‘normalisation’ practices [42: 59, 43], monster theory has significance in ageing and dementia research to scrutinise use of restrictive practices for dementia behaviour management within institutional care.

### Vulnerability is Challenging is Monstrous

Vulnerability is a central concept to law and policy about older adults with cognitive impairment [48, 49]. Conventional legal categorisation of a person or group as ‘vulnerable’ is usually associated with an inherent characteristic that exposes them to a risk of harm and often coupled with notions of ‘victimhood, deprivation, dependency, or pathology’ [48, 50: 8]. An alternative theory by Fineman is noteworthy as it recognises vulnerability will inevitably arise in all people from time to time based on fluctuating needs and circumstances [50].^15^ As an established identifier, vulnerability arouses pity for ‘wounded’ deserving persons that engenders a ‘good to be good to’ moral response [53: 832]. In this sense, vulnerability is constructed as a weakness in need of protection that frames the foundation for social and legal interventions. Vulnerability is often correlated to a person’s status within a notional age group [48, 54], particularly during the COVID-19 pandemic [3, 55]. Older persons living with dementia, bearing both characteristics of age and cognitive impairment, are made ‘doubly’ vulnerable. However, this coupling of age, cognitive impairment and vulnerability may lead to persons living with dementia being ‘trapped in their categorization as a vulnerable group’ [48: 56, 51: 142], and makes visible the ‘burden’ of their vulnerability [56] that may contribute to ageism and ‘benevolent othering’ [55, 57]. For older persons living with dementia, frailty and dependence may be conflated with, and taken as evidence of, incapacity that informs paternalistic legal decisions on a ‘status or characteristic-based vulnerability’ approach [48: 58, 58].^16^ Constructions of vulnerability, although sympathetic, are revealed to cast a shadow of lesser citizenship because the ‘vulnerable do not possess themselves and therefore, must be reinvented as dependent relative to those who are whole and healthy’ [60: 401].

At the same time, ‘challenging’ is a concept representative of abject bodies not easily managed or contained, such as female, disabled and queer bodies, and more recently, those with dementia. Abjection theory, with origins in psychoanalysis and the work of Kristeva, points to the material and symbolic segregation of the ‘clean and proper body’ from the impure, unruly and disruptive of life and order [60–62]. Found in the transgressive, such as deviance, waste, disease and decay that threatens civilised categories, abjection has relevance for body, disability and dementia studies: while the abject must be repelled, it is never quite expelled, leaving ambiguity and discomfort for the ‘stranger that we despise but fear we might become’ [60: 406]. As an identifier, abjection arouses disgust for uncontrollable, unruly bodies that provokes a ‘good to mistreat’ moral response [53: 832]. The abject body is produced in persons living with dementia through degenerative processes that manifest in loss of memory, mobility and speech, incontinence and behaviours that threaten human autonomy and integrity [63: 232]. Dementia provokes abjection because it threatens (challenges) the ‘clean and proper [mind and] body’ and is more than, ‘lack of cleanliness or health … but what disturbs identity, system, order. What does not respect borders, positions, rules. The in-between, the ambiguous, the composite’ [61: 4]. Persons living with dementia are positioned as abject in defence to the conceptual and corporeal threat of ‘challenging’ dementia behaviours that embody the Other and are ‘judged to be repulsive such as wandering, agitation, assault, … Confronted by the defective mind of the Other, contamination becomes a permanent threat for integrity’ [63: 232]. Within institutional aged care, abjected bodies of persons living with dementia in aged care with ‘unmanageable’ dementia behaviours are framed as ‘challenging’, ‘agitated’, ‘violent’ and ‘aggressive’ [64–67]. This is consistent with dominant approaches in dementia care that focus primarily on ‘problematic behaviours and undesirable functions of the body’ to ‘manage “challenging behaviours” with mechanical, environmental and/or pharmacological/chemical restraints’ [68: 3].

Drawing these literatures together, concepts of vulnerability and challenging are shown to both point to Shildrick’s ‘monstrous leaky bodies’ that defy categories and threaten norms [69]. Monstrous theory has been widely applied in disability, feminist and ageing scholarship to describe bodies that are ‘unbounded, leaky, fragmented and lacking control’ [60, 69, 70: 12, 71], and more recently, associated with dementia studies [72–76]. Behuniak acknowledges the ‘easy slippage between monsters and people’ living with dementia based on zombie characterisations of appearance, loss of self and that ‘death is preferable’ [77: 72, 84]. Dementia has been described as ‘refiguration’ of the monstrous, where the Other is constructed to reside not only in bodily difference but also in internal ‘unknowable, uncontrollable future[s]’ [72: 83, 73: 6]. This unfathomability is captured in an account of caring for a person living with dementia: it is ‘hard because he has good periods, when he is nice and gentle and we can sit down and talk and suddenly he is like a monster’ [63: 235 quoting, 78: 6].

The figure of the monster is thus revealed in inspection of co-constructions of ‘vulnerable’ persons with dementia who exhibit ‘challenging’ dementia behaviours, that:

…remind[s] us is that the monstrous is about embodiment as much as subjectivity… The paradox is, as with the monstrous or abject, that the ageing embodied subject in this way both comes to embody difference(s) and to challenge the idea that such differences are given [72: 71, 86].

In this way, dementia behaviours are constructed as the embodiment of the monstrous challenging Other in vulnerable persons living with dementia that represent disruption of both material and symbolic normative ageing futures.

### Monstrous Bodies ‘Wander’

While persons living with dementia may exhibit a broad range of behaviours, movement by people with dementia (known as ‘wandering’) is of particular concern to caregivers, health professionals and care institutions [66, 79, 80]. ‘Wandering’ is problematised movement by the pathologised body with dementia:

Walking is understood to be possible when the mind and body work together correctly, while wandering is perceived as when the mind is ‘lost’ and the body takes over [79: 280].

Characterised as purposeless, ‘wandering’ is the ‘wrong kind of walking’ that signifies a ‘literal and figurative “losing oneself”’ [79: 282, 271].^17^ Indeed, monstrous constructions are evoked in the ‘slow shuffle … relentless walking’ by persons living with dementia, prompting zombie-like characterisations as the ‘walking dead’ [77: 79]. Where monsters are identifiable by their behaviour [46: 134], ‘wandering’ is constructed as a signifier of monstrousness. In this sense, the monster is both produced and embodied in ‘wandering’ dementia behaviours that are ‘self-fulfilling in the social imaginary’ as proof of mindlessness and irrationality [79: 282] in persons who are ‘*always already* a danger to self and others’ [81: 211, emphasis original].

Biomedical models of dementia frame ‘wandering’ as an aimless risky pathology associated with harm and getting lost that requires surveillance and prevention strategies [79–83]. Within institutional care, ‘wandering’ by persons living with dementia is framed as ‘challenging to care practices’ and a threat to safety to themselves, others and institutional structures of aged care [66, 79: 270, 80]. However, person-centred and embodied personhood approaches allow for meaning consistent with a person’s prior hobbies [66] or ‘having a world’ [81: 222 quoting, 84], while persons living with dementia express enjoyment and health benefits [83, 85]. Dementia scholarship reveals ‘wandering’ by persons living with dementia to be a contested dementia behaviour that provokes management strategies for control and containment.

### ‘Last Resort’ Emergency Regulation

‘Last resort’ strategies for management of dementia behaviours within institutional aged care can be seen as practices that respond to the threat of the monstrous challenging Other. Once rendered monstrous, the body becomes the legitimate object of management and regulation practices:

By focusing on the individual disabled body, bodies are seen as vulnerable and dependent and are thus precarious in their perceived need to be controlled, warehoused, and regulated. If residents are classified as vulnerable, weak, or victimized, powerful decision-makers are more likely to see them as the [O]ther [86: 81].

Vulnerable persons living with dementia in institutional care, having been marked monstrous with dehumanising descriptions of pathologised dementia behaviours, such as ‘wandering’, ‘challenging’, ‘disturbing’ or ‘disruptive’, stimulate a ‘crisis management’ response [67]. Crisis constructions of dementia behaviours as an emergency threat to safety, security and order necessitate ‘ways to prevent, correct, or otherwise manage the unruly and vulnerable bodies of ageing humans’ within aged care [72: 78]. ‘Last resort’ restrictive practices in institutional aged care are consistent with crisis management of ‘challenging’ dementia behaviours that aim to restore normative control and regulation. Moreover, this crisis framing casts persons living with dementia themselves as the ‘emergency’ under threat of their inherent vulnerability that legitimise coercive practices [71: 185].

On the basis the ‘monster is an exception that suspends the law’ [42: 66, 43, 44, 87], restrictive practices for ‘emergency’ management of monstrous bodies of persons living with dementia can be understood as extra-legal ‘normalising responses’:

It is not by chance that the human monster signals a crisis … And it is not by chance that this crisis is resolved through the very body of the monster that becomes an object of sacrifice, of persecution practices [42: 57].

Medico-legal forms of control can be characterised as legitimised ‘curative violence for a curative futurity’ in relation to monstrous bodies [71: 180], that take expression in authorised use of physical and chemical restraints for dementia care practices within institutional care. In the next section this literature on monstrous constructions of vulnerable people with challenging bodies will frame analysis of the Case Study. This approach explores the production of monstrousness in Mr Reeves’ ‘wandering’ and dementia behaviours by the Australian aged care system that was (re)produced and reinforced by the RCAC to authorise normalisation practices of physical and chemical restraint in aged care.

## Dementia Behaviours and Restrictive Practices: The Garden View Case Study

Before examining the Case Study itself, this section provides a brief summary of the RCAC’s approach to dementia behaviours and restrictive practices. The RCAC inquired into the issue of restrictive practices in dementia care during the May 2019 Sydney Hearings. Two briefing papers on dementia and restrictive practices in institutional aged care were prepared by staff of the Office of the RCAC for the Commissioners and Public as background information to the Hearings [15, 88]. In the RCAC briefing papers, dementia behaviours were described as: ‘challenging behaviours, or behaviours of concern’ that ‘may include agitation or extreme restlessness, physical and verbal aggression, wandering, social and/or sexual disinhibition, delusions, apathy, depression and/or anxiety…’ [88: 7]. Restrictive practices were defined as ‘activities or interventions, either physical or pharmacological, that have the effect of restricting a person’s free movement or ability to make decisions’ that operate within aged care as ‘practices that control the behaviour of a resident, which may occur with the intention of reducing risks to a resident or others’ [15: 2]. Restrictive practices were acknowledged to be ‘often used on people with cognitive impairment who exhibit challenging behaviour, including people exhibiting the behavioural and psychological symptoms of dementia’ [15: 8].

Volume 4A of the RCAC Final Report presents several case studies heard during the Sydney Hearing that focused on issues related to dementia care within institutional aged care. This paper focuses on the Garden View Case Study (Case Study) that investigated the application of restrictive practices to manage Mr Reeves’ dementia behaviours at Garden View Nursing Home.^18^ The main facts of the Case Study are set out in the boxed text below:

### Garden View Case Study

#### Mr Reeves’ Garden View Admission

Mr Terance (Terry) Reeves is a married man with three children who was born in 1946. He was diagnosed with dementia in 2010. When his wife, Mrs Lillian Reeves, planned to travel overseas she sought respite residential care for Mr Reeves at the Garden View Nursing Home (Garden View). Mr Reeves stayed at Garden View between 1 May and 7 July 2018, a period of 67 days. At that time, more than 80% of Garden View residents had a recorded diagnosis of dementia. Mr Reeves’ aged care assessment recorded advanced Alzheimer’s Disease, some dementia behaviours and recommended specialised dementia accommodation. From the start of his admission to Garden View, Mr Reeves was noted to be ‘unsettled’ and exhibit ‘wandering’ behaviour. Mr Reeves was frequently placed within the ‘secure’ dementia unit. Garden View commenced use of chemical restraints on Mr Reeves on 5 May 2018 and physical restraints on 8 May 2018, which continued for the remainder of his admission. As time went on, Mr Reeves’ wellbeing deteriorated, and he experienced several falls. Mrs Reeve removed Mr Reeve from Garden View on 7 July 2018.

#### Regulator Findings

The aged care regulator, the Aged Care Quality and Safety Commission (ACQSC), reviewed Garden View in January 2019 and found Garden View had failed to meet accreditation standards and had placed Mr Reeves at serious risk. ACQSC did not make any findings against Garden View of harm or mistreatment of Mr Reeve.

#### RCAC Findings

The RCAC made findings that Garden View had applied physical restraints to Mr Reeves for multiple aggregated hours most days of his 67-day admission, including up to 14 hours on at least five days, and had administered psychotropic medication to Mr Reeves without lawful informed consent. The RCAC concluded Garden View had made frequent and extended use of physical and chemical restrictive practices that amounted to substandard care, however, declined to make findings of unlawful confinement or mistreatment of Mr Reeves, nor that excessive use of restraints had caused or contributed to his deconditioning.

This paper traces a line of monstrous constructions of Mr Reeves’ dementia behaviours in the Garden View Case Study that runs from witnesses’ evidence and written documentation presented to the RCAC to the RCAC’s own commentary and findings. Conducting an analysis of the ways in which the Case Study’s language and narratives discursively construct persons living with dementia and dementia behaviours, this approach identifies monstrous stories about the ‘challenging’ bodies of ‘vulnerable’ people in institutional aged care told to, and by, the RCAC. In the Case Study, the RCAC failed to resist discursive production of persons living with dementia as ‘vulnerable monsters’, instead promoting an ‘authorisation’ narrative about use of restrictive practices on persons living with dementia. Drawing out the RCAC’s (re)production of monstrous constructions of dementia behaviours discloses the normalising work performed by restrictive practices that legitimise medico-legal frameworks of control within institutional aged care.

## Monstrous Narratives in the Garden View Case Study

### ‘Challenging’ Monstrous Dementia Behaviours

This part examines the Case Study to show how its persistent construction of Mr Reeves’ dementia behaviours as ‘challenging’ was representative of the monstrous abject Other. This discursive construction occurred through recurrent use of terms such as ‘behavioural issues’, ‘aggression’, ‘unsettled’, ‘disruptive’, ‘agitation’ and ‘confusion’ that presented an institutional ‘challenge’ for Garden View. This is consistent with Mr Reeves dementia behaviours being ‘filtered through the lens of pathology’ that ‘characterises behaviours as challenging’ [67: 162].

An unbroken line of monstrous construction of Mr Reeves’ behaviour, particularly his ‘wandering’, can be traced through agents in the aged care system, including government assessors, aged care staff and medical practitioners, to its adoption and (re)production by the RCAC. From Mr Reeves’ entry into the aged care system, his dementia behaviours were conceptualised as a ‘challenging’ threat to institutional structures of care and reflective of understandings of monsters as strangers who come from outside to disrupt safety [46: 131]. The Aged Care Assessment Team (ACAT) assessment recorded Mr Reeve’s had advanced Alzheimer’s Disease, limited ability to communicate, ‘experienced some aggressive incidents and wandering behaviour’ and required the ‘skills and contained environment of a specialized dementia unit’ [11: 80]. Upon his admission to Garden View on 1 May 2018, Mr Reeves was assessed by the visiting medical practitioner as requiring ‘normal nursing care … tends to wander around’ [11: 82]. That evening, on Mr Reeves’ first night in Garden View, the progress notes record ‘resident remains awake and wandering’, while the ‘LMO communication book’ for the visiting medical practitioner noted: ‘Terance Reeves – unsettled, wandering +  + ’ [11: 84–85]. One week later on 7 May 2018, medical notes record Mr Reeves’ was ‘wandering a great deal’, ‘generally unsettled’ and was prescribed psychotropic medication ‘as required for “Behaviour/Unsettled”’. The treating medical practitioner documented this as ‘… Resident was extremely agitated, confused and wandering extensively’ [11: 88].

During the RCAC hearing, Garden View’s Director of Nursing gave evidence that physical restraints were used at Garden View on residents with ‘challenging behaviour’ [11: 91]. Following investigation into Garden View’s use of physical restraints, the Aged Care Quality and Safety Commission concluded Garden View had placed Mr Reeves’ ‘safety, health or wellbeing at serious risk by failing to manage his challenging behaviours’ [11: 106]. These examples demonstrate the aged care system’s persistent pathologisation of Mr Reeves’ dementia behaviours in ‘challenging’ language — terms such as ‘wandering’, ‘excessive’, ‘agitation’, ‘unsettled’, ‘confusion’ and ‘aggressive incidents’ — that constructed him as a transgressive Other within the aged care system. In challenging the ‘natural order’ at Garden View, Mr Reeves’ dementia behaviours represented a threat to ‘safety’ at Garden View that required practices of containment and normalisation.

The RCAC reflected and reinforced monstrous constructions of dementia behaviours in the Case Study by using language that (re)produced ‘challenging’ discursive terms such as ‘wandering’, ‘aggression’, and ‘unsettled’. In adopting the ‘challenging’ lexicon used by the aged care system to describe Mr Reeves’ dementia behaviours, the RCAC did not contest monstrous constructions of older persons whose bodies do not fit cleanly or neatly into institutional care. This tacit approval of monstrous language appears in the RCAC’s description of Mr Reeves’ behaviour on the first night as ‘very unsettled from the outset’ and ‘wandered at night unless diverted’ [11: 83]. The RCAC goes on to adopt ‘security crisis’ terminology in reporting that, within five days of entering aged care, Garden View’s ‘“red alert” monitoring chart’ for ‘residents who wander a lot’ led to Mr Reeves being ‘closely monitored’ and often confined to a ‘secure’ dementia area where people were either ‘bedridden or restrained’ [11: 87]. In reporting events leading to Garden View’s first use of physical restraints on Mr Reeves, the RCAC again endorsed ‘challenging’ language in ‘disruptive behaviour’ and ‘naked intrusion’ [11: 92]. Although the RCAC distanced itself from equating Mr Reeves’ ‘non-cooperation’ with Garden View staff to aggression, it did not interrogate constructions of dementia that attribute it as cause of his ‘aggression’ and the legitimate target of individualised behaviour modification strategies such as physical restraint [64, 65]. Similarly, the RCAC replicated ‘challenging’ language of ‘wandering’, ‘agitat[ion]’, ‘confusion’ and ‘distress’ in reporting the treating medical practitioner’s decision to increase the dose of chemical restraint for Mr Reeves. On the basis Mr Reeves’ was ‘not settling’, ‘wandering around’, ‘getting agitated with staff’, ‘in some distress’ and ‘in a confused state’, dementia was positioned both as the reason for his ‘challenging’ behaviour and the premise of restrictive practices in his ‘best interests’ and the ‘sake of safety’ [11: 97].

In accepting the dominant framework within institutional aged care of ‘assessing, correcting and controlling’ dementia behaviours to prevent harm [67: 171], the RCAC did not dispute the role of restrictive regimes to manage ‘challenging’ monstrous bodies of persons with dementia. Further, nor did the RCAC question the role of ‘secure’ locked dementia units as a ‘containment’ strategy within institutional care. This conceptualisation reveals figuration of the monster in Mr Reeves’ dementia behaviours that compels a control response:

… through definitions, classifications, distinctions, the discourse of the monster produces its object, constructs continually its specific monster in order to then deliver it to the practices of exclusion or normalization [42: 59].

In endorsing this ‘challenging’ frame for Mr Reeve’s dementia behaviours, the RCAC’s language pathologises behaviours of persons with dementia that provokes a ‘crisis management’ response within institutional aged care [67] and is consistent with conflation of dementia with threat, in what Graham calls the ‘securitisation of dementia’ [80].

### ‘Wandering’ as Embodied Monstruous

This part follows construction of movement by Mr Reeves from evidence presented to the RCAC, to its reflection and reproduction by the RCAC as aimless and irrational, and problematised as dehumanised ‘wandering’ in the Garden View Case Study. This approach acknowledges health and safety concerns in dementia care at the same time as unpacking constructions of ‘wandering’ as a dementia behaviour for institutional care ‘to assess and manage’ with medication and restraint strategies [66: 736].

It is clear from the Case Study that Mr Reeves’ ‘wandering’ was the most ‘challenging’ of his dementia behaviours for the aged care system to manage. Mr Reeves’ movement ‘challenged’ structures of aged care consistent with a monster’s ‘potential to contaminate and undermine systems of order’ [46: 127]. In this way, Mr Reeves’ ‘wandering’ both produced *and* embodied his monstrousness. Agents in the aged care system regarded Mr Reeves’ movement as evidence of his incapacity, framing it in dehumanised ‘walking dead’ language of ‘wandering’ that discursively located it outside the human realm [77]. The monstrous figure of the zombie emerged from ACAT’s assessment that Mr Reeves’ needed a ‘contained environment’ due to his ‘wandering behaviour’ [11: 80]. The figure was present in Garden View’s progress notes that Mr Reeve ‘tends to wander around’, ‘remains awake and wandering’, ‘[is] unsettled, wandering +  + ’, and ‘wander[s] a great deal’ [11: 82, 84, 85, 88]. Lastly, the figure appears in evidence of the treating medical practitioner about ‘charting’ antipsychotic medication because Mr Reeves ‘…was extremely agitated, confused and wandering extensively’ [11: 88]. Mr Reeves’ ‘wandering’ was conceptualised by the aged care system as a monstrous ‘challeng[e] to care practices … something threatening that need[ed] managing’ with physical and chemical restraints [79: 270].

In the Case Study, the RCAC reported Mr Reeves’ movement as ‘wandering’ in multiple passages, such as ‘he wandered at night unless diverted by nursing staff, then was drowsy during the day as a result of day-night reversal’, ‘Mr Reeves was unsettled and wandering a lot’ and ‘wandering was creating a risk of falling’ [11: 83, 85, 97]. The RCAC reproduced monstrous terminology and, in doing so, failed to reject the figure of the ‘wandering’ monster that reflect assumptions Mr Reeves’ walking lacked rationality or meaning. The RCAC’s repeated use of the non-preferred term ‘wandering’ perpetuated conceptions of dehumanised movement by persons living with dementia, where “‘wandering’ is in and of itself evidence of the no longer mindful state of someone with dementia’ [79: 282].^19^

### Restrictive Practices ‘Last Resort’ Response

Within institutional aged care, regulated use of physical and chemical restraints — positioned as ‘last resort’ or ‘emergency’ measures — operate as extra-legal exceptions to ‘manage’ dementia behaviours and prevent harm. This part investigates the Case Study for how restrictive practices can be viewed as extraordinary ‘normalising practices’ to the constructed ‘emergency’ of Mr Reeves’ behaviours at Garden View, and the ways this was (re)produced by the language of the RCAC.

Garden View’s policy manual defined physical restraint as the ‘intentional restriction of a person’s voluntary movement or behaviour by the use of a device or physical force for behavioural purposes’. It stipulated physical restraints were only to be applied as a ‘last resort … where all other alternatives have been determined as ineffective and/or inappropriate’ [11: 83, 102]. In fact, Garden View’s unease about its own use of restrictive practices is evident in an internal email advising staff ‘residents in the central lounge if need to be restrained, please sitting [sic] them near the glass door side, it doesn’t look nice when the visitors walk in and see resident been [sic] restrained’ [11: 91]. The Garden View consent form completed for Mr Reeves indicated restraint was required due to ‘danger to self and others’ and would be applied ‘under supervision and recommendation of Registered Nurse’ [11: 95]. Restrictive practices are intended to be a ‘when-all-else-fails’ behaviour management strategy within institutional aged care, as expressed in the medical practitioner’s evidence about administration of chemical restraints on Mr Reeves, ‘these sorts of medications are really last resort medications, and you don’t go flying into it straightaway’ [11: 98]. However, the Case Study does not contain evidence of danger or harm caused by Mr Reeves, pointing to the ambiguous nature of the ‘emergency’ his ‘wandering’ presented.^20^ This was acknowledged in evidence to the RCAC, when Garden View’s Director of Nursing denied breach of the restraints policy on grounds ‘[it] says that he can be restrained under emergency basis, but then it is very hard to demonstrate what is emergency basis’ [11: 102]. In resorting to the use of restrictive practices, Garden View located the emergency, based on an *anticipation* of danger and harm, in Mr Reeves’ dementia. Constructing dementia itself as the threat ‘naturalises violence’ in ‘aberrant individuals’ with dementia and rationalises use of restrictive practices and segregation in institutional care [64: 2085–2086]. This is similar to constructions of persons with disability themselves as an ‘emergency’ that permits pre-emptive forms of coercive control [71]. Indeed, restrictive practices are acknowledged to be used to regain order and control of ‘challenging’ abject bodies in institutional aged care [63: 236]. This is also consistent with findings that aged care discourse positions dementia as the primary cause of ‘crisis’ in a way that minimises structural aged care issues that contribute to dementia behaviours [65].

In reporting Garden View’s frequent and extended use of restraints on Mr Reeves, the RCAC did not dispute the role of ‘last resort’ restrictive practices within an institutional framework of dementia behaviour management strategies. The RCAC did reject the argument that physical restraints had been validly applied to Mr Reeves in circumstances constituting an emergency on the basis Garden View had not exhausted alternative management strategies [11: 96, 103].^21^ However, the RCAC did not interrogate the anticipation of danger and harm constructed by Garden View in Mr Reeves’ dementia behaviours that activated and allowed for a cascade of behaviour management responses. The RCAC accepted a crisis framing of Mr Reeves’ ‘wandering’, co-constructing dementia with emergency in examples such as ‘red alert monitoring chart’ and ‘restraint would be applied only as a last resort for Mr Reeves’s safety’ [11: 87, 104] that sanctioned a regime of normalising practices, culminating in physical and chemical restraints. This approach discloses construction of bodies with dementia within the aged care system as monstrous Others who ‘must be re-contained by strategies of normalisation [such as] institutionalisation’ [89: 12]. In failing to dispute monstrous constructions of dementia behaviours that legitimise extra-legal responses, the RCAC reflected understandings that ‘ordinary measures against monsters will not succeed… something extraordinary is required to resolve monsters…’ [46: 136]. In this sense, ‘last resort’ restrictive practices represent an authorised normalising response to the monstrous threat of risky dementia behaviours within institutional aged care.

## RCAC ‘Authorisation’ of Normalising Practices

In addition to adopting language of ‘last resort’ restrictive practices within institutional care, the RCAC promoted an ‘authorisation’ narrative focused on written procedures, informed consent, legal validity and clinical justification. The RCAC found Garden View’s use of restrictive practices on Mr Reeves was not an authorised ‘last resort’ emergency response based on noncompliance with its own internal procedures. The RCAC emphasised ‘authorisation’ when it reported:

In the period May to July 2018, the written policy of Garden View concerning the use of restraints was that they could be used only as a last resort and with the written authorisation of both the resident’s medical practitioner and the authorised representative of the resident … There was no authorisation of any kind in place for physical restraint to be applied to Mr Reeves, and no record of this use of restraint was made in a restraint chart, progress notes, or any other record produced by Garden View to the Royal Commission.[11: 92].

The RCAC’s ‘authorisation’ narrative was further developed in its regulatory focus on policy and procedures, such as ‘application of physical restraints without prior consent or authorisation’, ‘restraint authorisation form’, ‘form authorising physical restraint’, ‘restraint chart’ and ‘protocol for the use of restraints’ [11: 94, 101, 102]. The RCAC limited its inquiry in the Case Study to whether restraint of Mr Reeves had been properly regulated — with a focus on compliance with Garden View’s internal written policy — that would have legitimised ‘authorised’ restrictive practices. Despite finding Garden View’s actions were not ‘authorised’ due to noncompliance, the RCAC’s approach raises the question that had Garden View complied with its written policy, would its ‘frequent’ (almost daily) and ‘extended’ (up to 14 hours per day) use of restrictive practices on Mr Reeves have been an ‘authorised’ (acceptable) use of restraints? While the RCAC made findings of substandard care in Garden View’s frequent and extended use of restraints without consent, it declined to find this amounted to unlawful confinement or mistreatment, nor that use of restraints caused or contributed to Mr Reeves’ deconditioning. The RCAC’s findings permitted extraordinary restraint practices on a person living with dementia within institutionalised aged care consistent with extra-legal normalising responses to monsters:

Their elimination or integration through legal means are not so much legal treatments of those individuals qua monsters as they are means or abstracting their monstrosity away. Disabled people can indeed be ‘normalized’, i.e. made ‘normal’, same, ‘one of us’. … Insofar as they can be normalized, they are no longer ‘monsters’ since legal norms can apply to them, at least partially. This normalized portion of themselves is entitled to legal personhood, though their legal prerogatives and rights may only be partial [43: 312, 313].

In concentrating on ‘authorisation’ of restrictive practices, the RCAC contemplated an extra-legal regime of ‘last resort’ physical and chemical restraints that tolerated ‘elimination or integration’ practices to normalise monstrous bodies within institutional aged care.

Explicating monstrous narratives in the Case Study, this paper has demonstrated the ways the RCAC repeated and perpetuated constructions of dementia behaviours that authorise normalising restrictive practices to ‘de-monster’ persons living with dementia. In this sense, physical and chemical restraint practices represent an extra-legal response to monsters that operates ‘outside-law, or [in] violence … or medical cures’ [42: 66]. Moreover, beneath its ‘authorisation’ narrative, lies an avoidance by the RCAC to confront the figure of the monster in constructions of dementia behaviours that permit an ‘authorised’ suspension of the law. While the RCAC made significant recommendations in its Final Report for improved dementia care, increased staff training in dementia and stronger regulation of physical and chemical restraints, it failed to reject the fundamental normalising role of restrictive practices and coercive control within institutional aged care. Drawing on disability, feminine and dementia scholarship, this paper has argued that while regulatory legal and clinical frameworks authorise restraint practices to protect vulnerable persons, they also serve as normalising responses to control the challenging monstrous Other. In failing to resist monstrous constructions of dementia behaviours, the RCAC accepted narrow medico-legal approaches for the management of unruly leaky bodies that do not fit neatly into institutional care and perpetuated ambivalent law and policy in this area. Institutional use of restraints is highlighted as a contested dementia care practice for management of ‘vulnerable’ people with ‘challenging’ behaviours. Restrictive practices for dementia behaviours seek to ‘civilise’ the monstrous body as it ‘forgets’ and ‘fails to remember how it is contained, becoming [O]ther … [yet] Holding such a body becomes in the end: an impossibility’ [62: 10]. The (re)appearance of monstrous language in the RCAC suggests a reluctance to refute monstrous constructions of persons living with dementia and the normalising role performed by restrictive practices in institutional aged care.

## Conclusion

This paper has revealed (re)production of constructions of persons living with dementia as ‘vulnerable monsters’ in the discourse of the RCAC in its Final Report. Narrative analysis of the Garden View Case Study disclosed the telling of a dementia ‘monster’ story by the aged care system that was retold and reinforced by the RCAC. While older persons living with dementia were categorised by the RCAC as a vulnerable group that required protection, this same vulnerability also framed constructions of dementia that legitimised restrictive practices in dementia aged care. Drawing on dementia, disability and feminist theory about unruly and leaky bodies, extracts from the Case Study frame dementia behaviours as ‘challenging’ in language such as ‘aggressive’, ‘violent’ and ‘disruptive’ that are consistent with descriptions of the monstrous Other. Further, repetition of the term ‘wandering’ produced movement in persons living with dementia as dehumanised, problematised walking that signified the embodied Other. Persons living with dementia are co-constructed as ‘vulnerable’ to degenerative symptoms, getting lost and injured and as a ‘challenging’ threat to others and the aged care system through a ‘language of pathology’ [80: 442]. Once constructed as monstrous, ‘wandering’ and other dementia behaviours require and legitimise an emergency crisis response in the form of ‘last resort’ chemical and physical restraints. This analysis has explicated an unexpected association between vulnerability and monstrousness that underpins medico-legal forms of control for older persons living with dementia in aged care. This paper has argued that in failing to resist monstrous constructions of dementia behaviours, the RCAC tolerated and authorised an extra-legal regime of restrictive practices to normalise unruly leaky bodies in aged care. Although dementia care and restrictive practices received substantial attention in the RCAC, this paper reveals a missed opportunity for deeper review of institutionalised use of restraints that has relevance for ongoing reform of Australian aged care following conclusion of the RCAC.

## Footnotes

1 This vulnerability theme emerged strongly during the COVID-19 pandemic in media headlines and reporting, such as ‘Aged care in Australia—why has the system failed our vulnerable old people?’[1] and ‘people living with dementia are one of the most vulnerable groups in society at this time’ [2]. For a detailed analysis of media framing of older people and vulnerability during COVID-19, see [3].

2 In this paper, the phrase ‘dementia behaviours’ is used in preference to other terminology, such as ‘adverse behaviours of dementia’ or ‘behavioural and psychological behaviours of dementia’ etc., which may be pejorative and stigmatising, see [4, 5]. However, the author acknowledges that social acceptance of words evolve and meanings ascribed will vary between persons; what was once considered dehumanising may come to be (re)claimed by the subject user, see for example ‘senile’ [6] and ‘demented’ [7].

3 In the RCAC Final Report older people living with dementia in aged care were associated with vulnerability, see for example: ‘at all times care should be respectful, engaging and kind. Indeed, it is precisely when a person in need of care has a severe cognitive impairment or some other vulnerability that it is most critical to stress the rights of that person’ [8: 7], ‘many, if not most, approved providers care for people who are very vulnerable, including due to frailty, dementia and cognitive impairment’ [9: 506], ‘aged care nursing is a lot more demanding, with incontinent people, people with dementia, people with very serious—and vulnerable people’ [10: 602].

4 Dementia behaviours were framed in the Final Report as ‘challenging’ to manage, see for example: ‘case studies at this hearing illustrated that managing behavioural and psychological symptoms of dementia can be a challenge for aged care providers. In addition to the case studies, we heard from Australian experts in research into dementia and into measures taken in residential care in response to behaviours seen as “challenging”’ [11: 70], and ‘Mr UI was living with dementia [and] displayed “challenging aggressive behaviours” … Ms Sargent gave evidence that despite Mr UI’s challenging behaviours …However, Mr UI’s behaviours continued to present challenges for the staff at Pioneer House. These challenges were reported to the board in a series of reports…’ [10: 598].

5 The Australian Government reports 54% of persons living in permanent residential care have a diagnosis of dementia [14], however the proportion is likely much higher at approximately 70% [11: 67].

6 There is lack of data on prevalence of restrictive practices in aged care, although estimates suggest psychotropic medication is administered to approximately two-thirds of aged care residents, while physical restraints may be used on up to 40% [15: 11]. Disclosed data from an individual aged care provider revealed chemical restraints were administered to 71% and physical restraints applied to 50% of residents [16: 97].

7 The Final Report of the RCAC spans eight volumes: Volume 1 provides a summary of the full report and all 148 recommendations; while restrictive practices were examined in depth in Chapter 3 of Volume 3A and dementia care in Chapter 3 of Volume 4A.

8 Over many decades a series of more than 30 major reviews and inquiries into the aged care sector have recommended reform of both the quality and models of aged care in Australia, see [20, 21].

9 The RCAC also recommended that following conclusion of the Royal Commission into Violence, Abuse, Neglect and Exploitation of People with Disability that regulation of restrictive practices be alligned across aged care and disability services [8: 110].

10 The reforms respond to 17 recommendations of the RCAC and are designed to deliver improved quality and safety of care and greater transparency and accountability of aged care providers under the *Aged Care and Other Legislation Amendment (Royal Commission Response) Act 2020,* for more detail see [23].

11 From September 2021, amendments to the *Aged Care Act 1997* and *Quality of Care Principles 2014* require aged care providers to have a clinical governance framework to minimise use of restrictive practices that documents attempted alternatives, regular monitoring for adverse effects, review for removal of restraints as soon as possible, together with an appropriate behaviour support plan. Further amendments under the *Aged Care and Other Legislation Amendment (Royal Commission Response) Act 2022* provide for a hierarchy of persons or authorised bodies for informed consent where ‘restrictive practices are necessary, and a consumer is unable to provide consent’ [24].

12 For a detailed examination of the history, practice and effectiveness of Royal Commissions in Australia, see [27].

13 Further related research has examined residential aged care facilities as sites of confinement for ‘disposable lives’ during COVID-19 [39], or ‘zones of neglect’ that negate responsibility [40], as well as ‘consumer choice’ as the primary measure of quality in aged care services [41].

14 While the RCICSA rejected the notion of the ‘paedophile monster’ in offenders themselves, Crofts argues monstrous institutional failures to prevent and respond to systemic child sexual abuse aroused a horror response [46]. Monster theory has also informed analysis of the New South Wales Wood Royal Commission Paedophile Inquiry [47].

15 Fineman proposes that ‘vulnerability’ can be conceptualised as ontological, as a universal or ‘lifecycle’ experience, common to all individuals albeit at different times in different ways due human corporeality and interdependence, for more see for example, [50, 51: 145, 52].

16 This ‘status-based’ classification of vulnerability has historically extended to other groups, such as women, gender minorities and people with disability, and can reinforce marginalisation and erode autonomy [59].

17 The term ‘wandering’ is also a contested term in autism spectrum disorder used to describe movement that is similarly perceived as lacking direction or destination and an aberrant behaviour [81].

18 The Garden View Case Study is a detailed examination of Mr Reeves admission to Garden View, based on written and oral evidence of Mr Reeves’ wife and family members, treating medical practitioners, nursing, care and management staff of Garden View, tender records, as well as that of Counsel Assisting the RCAC. In reporting the Case Study, the RCAC incorporates witness testimony, practitioner progress notes and Garden View documentation, in addition to its own commentary and findings. The Case Study is reported from pages 76 to 107 in Volume 4A of the Final Report.

19 Elsewhere in the Final Report, the RCAC did acknowledge the importance of avoiding ageist and stigmatising language in aged care, such as ‘toileting’ or ‘wandering’, that ‘position the older person as an object or a “job” that someone must complete’. The RCAC quoted evidence that terminology such as ‘“abscond, wander, BPSD [behavioural and psychological symptoms of dementia],” you know, these are people who are reacting to what’s going on around them. So those sorts of—that sort of language happens—matters. “The floor, feeding, toileting,” you know. How would you feel if someone said they were going to toilet you, not help you go to the bathroom?…it’s essentially very ageist and very—yeah very inhumane’ [16: 219].

20 The closest episode is reported as ‘…Mr Reeves had shown disruptive behaviour, including naked intrusion into other rooms, was “aggressive and put his fist in the air” … staff used the word “aggressive” when explaining the use of the restraints to Ms McCulla [Mr Reeves’ daughter]. When Ms McCulla asked whether this meant that Mr Reeves had tried to hit someone, she was told “no, he was yelling to stop it and he wasn’t cooperating”’ [11: 92].

21 The RCAC stated that ‘Garden View did not do everything that it could have done to investigate other options for managing Mr Reeves’s behaviours before imposing physical restraints on him’ such as seeking advice from the Dementia Behaviour Management Service or Severe Behaviour Response Team or implementing 1:1 care [11: 103].
